# A Detailed Numerical Model for a New Composite Slim-Floor Slab System

**DOI:** 10.3390/ma17071464

**Published:** 2024-03-22

**Authors:** Sławomir Dudziak, Paweł M. Lewiński

**Affiliations:** 1 Faculty of Civil Engineering, Warsaw University of Technology, Armii Ludowej Ave. 16, 00-637 Warsaw, Poland; 2 Building Structures, Geotechnics and Concrete Department, Building Research Institute (ITB), Filtrowa 1 Street, 00-611 Warsaw, Poland; p.lewins@wp.pl

**Keywords:** slim-floor system, steel–concrete composite beams, Abaqus, cohesive elements, concrete damaged plasticity model

## Abstract

The paper concerns the numerical modelling of a new slim-floor system with innovative steel–concrete composite beams called “hybrid beams”. Hybrid beams consist of a high-strength TT inverted cross-section steel profile and a concrete core made of high-performance concrete and are jointed with prestressed hollow core slabs by infill concrete and tie reinforcement. Such systems are gaining popularity since they allow the integration of the main structural members within the ceiling depth, shorten the execution time, and reduce the use of concrete and steel. A three-dimensional finite element model is proposed with all parts of the system taken into account and detailed geometry reproduction. Advanced constitutive models are adopted for steel and concrete. Special attention is paid to the proper characterisation of interfaces. The new approach to calibration of damaged elastic traction–separation constitutive model for cohesive elements is applied to concrete-to-concrete contact zones. The model is validated with outcomes of experimental field tests and analytical calculations. A satisfactory agreement between different assessment methods is obtained. The model can be used in the development phase of a new construction system, for instance, to plan further experimental campaigns or to calibrate simplified design formulas.

## 1. Introduction

**Figure 1:**
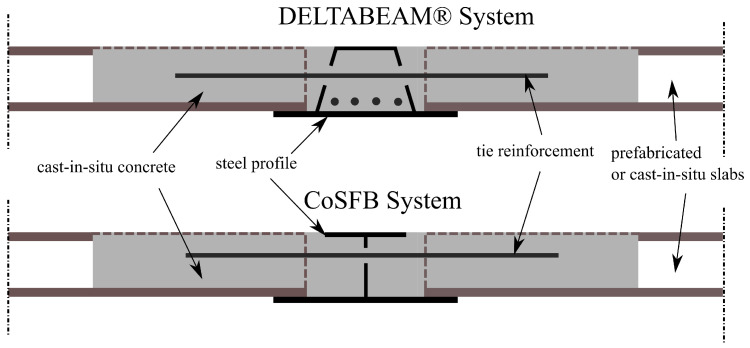
Examples of slim-floor systems.

In the case of public utility and industrial construction, steel–concrete composite structures have been commonly used for many years [1]. Such structures allow us to use the strength properties of both materials rationally, which plays an increasingly important role due to the requirements of sustainable development. Probably in the future, the interest in these structures will increase due to the easier availability of high-strength steel and concrete. Another advantage of this type of structures is that the construction process can be significantly shortened by using prefabrication technology. Among many types of steel-composite structures, slim-floor slabs are considered to be the leading innovative technology [2]. In such slabs, the main beams transferring loads to the supports are integrated within the slab’s depth. As a result, plain surfaces are obtained for both ceiling sides, which enables us to reduce the overall height of the building and to execute the installations easier. Many types of slim-floor systems are being developed, differing in the cross-section of the main beams or slabs supported on them, for instance ${\mathrm{DELTABEAM}}^{®}$ or CoSFB systems [3], see Figure 1.

### 1.1. Literature Review

Due to the great importance of steel-composite structures for the construction industry, many studies have been conducted on this topic all around the world. Hegger et al. [4,5] analysed through experimental studies the influence of flexible supports on the load capacity of hollow-core slabs installed in slim-floor ceiling systems. They concluded that the shear resistance of hollow core slabs in case of flexible supports should be reduced to 50–70% of the value calculated for rigid supports or special formulas should be used to determine this resistance (e.g., ”the Finnish model”). A similar topic was examined experimentally, analytically and numerically by Derkowski and Surma [6]. They also observed the decrease in shear resistance due to flexibility of supports can be up to 42% and connected this effect to uneven loading of ribs in hollow core slabs. Girhammar and Pajari [7] conducted research on the bond strength between prefabricated hollow core slabs and overlay concrete and revealed that a layer of concrete cast in situ can increase the shear resistance of slabs. Nardin and Debs [8] investigated the influence of shear studs and their arrangement on the stiffness and load capacity of the connection between beams and the slab part in slim-floor systems. They showed that without studs, this connection should be treated as a pinned one, and the use of the studs significantly increases the stiffness of this connection, and it can be classified as semi-rigid. Schaefer [3] formulated additional rules for checking steel beams installed in slim-floor systems. These formulas take into account e.g., the influence of the additional bending of the flanges in the direction perpendicular to the beam axis due to the support of slabs. Limazie and Chen proposed a non-linear numerical model in the ANSYS code for a slim-floor consisting of asymmetric steel I-beam and concrete cast on steel decks jointed together [9]. They used shell elements for steel profile with an elasto-plastic material model, continuum finite elements with fixed cracking constitutive model for concrete and contact elements for steel-to-concrete interfaces. Using such a validated model, they performed parametric studies concerning the thickness of concrete topping, the dimensions of openings in a steel profiles, and the web thickness. They formulated recommendations regarding the location and the size of openings in the steel part. Later, these authors [10] proposed analytical design formulas for the shear resistance of connections in slim-floor systems consisting of openings in steel profiles filled with concrete and reinforced with tie members. Yan and Liew [11] proposed formulas for the shear resistance of steel–concrete–steel sandwich plates based on experimental outcomes. Souza et al. [12] performed experimental and numerical tests of the shear resistance of a typical stud connection in slim-floor systems with hollow-core slabs. Based on the calibrated numerical model prepared in the Midas FX and Diana codes, they performed wide parametric studies concerning the yield strength of connectors and the class of concrete. They came to the following conclusions: code formulas (EC4 and NBR 8800) underestimate the load capacity of studs; the concrete class influences not only the load capacity of the studs, but also the distribution of forces between the studs; the impact of the studs yield strength is smaller for higher concrete classes. Lacki et al. [13] presented the results of engineering optimisation process of dowels shape in steel–concrete composite ceiling systems. They also compared the results of analytical and numerical calculations and formulated general guidelines for the dowels shape. Sheehan et al. [14] conducted an experimental testing campaign of 9 composite beams of slim-floor type, which differed in slab depth, load application conditions and method of connection. These tests were later simulated using the Abaqus software [15]. Their models took into account non-linear material models (Concrete Damaged Placiticity for concrete) as well as contact elements between concrete and steel (Coulomb friction). On the basis of these analyses, a general remark concerning the efficiency of concrete dowels and recommendations regarding their diameter were formulated. Albero et al. [16] analysed the fire resistance of various slim-floor systems using a validated FE model prepared in the Abaqus code. Farhan and Shallal [17] investigated the behaviour of innovative composite beams containing steel tubes filled with lightweight concrete and compared experimental outcomes for circular and square tubes. Kyriakopoulos et al. [18] prepared a numerical model of separate ${\mathrm{DELTABEAM}}^{®}$ for slim-floor solutions and validated it using laboratory tests results. They focused on the robustness and ductility of the tested beams and formulated some general remarks on the efficiency of various shear connections types. Alam et al. [19,20] analysed the fire resistance of slim floor beams through experimental and numerical tests. They formulated the following main conclusions: standard formulas (EC4) significantly underestimate the fire resistance of slim-floor systems, additional rebars located at the bottom of concrete core substantially improves the fire performance of such systems. Lukačević et al. [21] reported a new type of steel–concrete composite floor consiting of cold-formed steel profiles and cast-in situ concrete which is being developed within the LWT-FLOOR project. Finally, Lechman [22] performed a cross-sectional analysis to estimate a load capacity of separate hybrid beam.

Summing up the literature review, the topic of slim-floor systems is constantly arousing interest in many countries. Special attention is paid to the interaction between parts made of different materials as well as ensuring proper shear flow in connection between steel and concrete. Some strategies for numerical modelling of such systems using commercial finite element codes can be found in the literature, but they are usually limited to separate beams and do not cover the whole structural systems. Contact zones between steel and concrete are modelled using contact elements with Coulomb friction law in all references.

### 1.2. Research Significance

**Figure 2:**
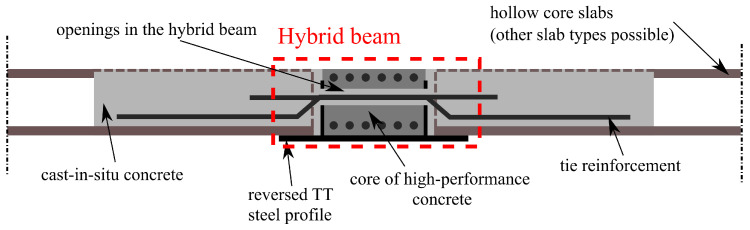
Scheme of the new slim-floor system analysed in the paper.

This paper concerns the new slim-floor system presented earlier by the authors, whose main designer is Mr Jerzy Derysz [23,24]. The essential novelty in this system is a new type of composite steel–concrete beam, the so-called hybrid beams (HBs). These beams include a reinforced concrete core made of high-performance concrete and a steel profile with an inverted TT cross-section, which cannot be found in other systems. HBs can be connected with different types of floors, for instance with hollow-core slabs or flat slabs cast in situ made of lightweight concrete [25]. The connection consists of openings in the HB, through which the tie reinforcement is passed, cf. Figure 2. The presented system has many advantages over other solutions. Firstly, separated hybrid beams are characterised by high torsional stiffness, which makes the slab execution easier since, unlike other systems, they do not require temporary supports during the assembly stage. Secondly, the compressive zone is much higher than in typical RC members, consequently, properties of steel and concrete of high strengths can be effectively used. Obviously, the presented system provides a shortening of the execution time and significant material savings.

Despite the rapid development of slim-floor solutions, papers on numerical simulations of their behaviour, in particular covering the whole structural systems, are rather rare, although with some exceptions [9,15]. Such simulations can be very useful at the stage of a new construction product development since they enable the reduction of time-consuming and expensive experimental research and help to calibrate the simplified formulas used in practice for dimensioning. During the preparation of numerical models, the verification and validation stage is essential [26], especially in the case of non-linear analysis. In this paper, a detailed 3D numerical model for a new slim-floor system is proposed and a process of its validation is presented. The model is prepared in the popular FE environment—the Abaqus program [27] (version 2018). Special attention is paid to the modelling the interfaces between different parts of the structural system. The main assumptions for this issue have been described earlier in the paper [28]. It is based on cohesive elements, which can more accurately reproduce the behaviour of concrete-to-concrete interfaces. The results obtained using the numerical model are confronted with the outcomes of full-scale experimental research and analytical calculations. Therefore, this paper presents a reliable calibration strategy for FE numerical models for entire slim-floor systems and a comparison of different approaches to estimation the load-bearing capacity of these systems are presented in this paper.

## 2. Field Experimental Tests

The extensive experimental testing campaign has been already described in previous papers by the authors [23,24,29]. Therefore, only results important for this numerical study are briefly summarised in this paper.

The tested slab consisted of two kinds of prefabricated elements: a hybrid beam BH 20–300 with a theoretical span of 5.80 m and prestressed hollow core slabs HC 200 of 6 m length and 200 mm depth supported on the flanges of the hybrid beam. The floor slab was integrated with the system of rebars perpendicular to the BH beam grouted in joints (see Figure 2). The in situ concrete of C20/25 class was cast into the gaps between prefabricated elements and into the keyways in the HC slabs, thus creating RC dowels.

**Figure 3:**
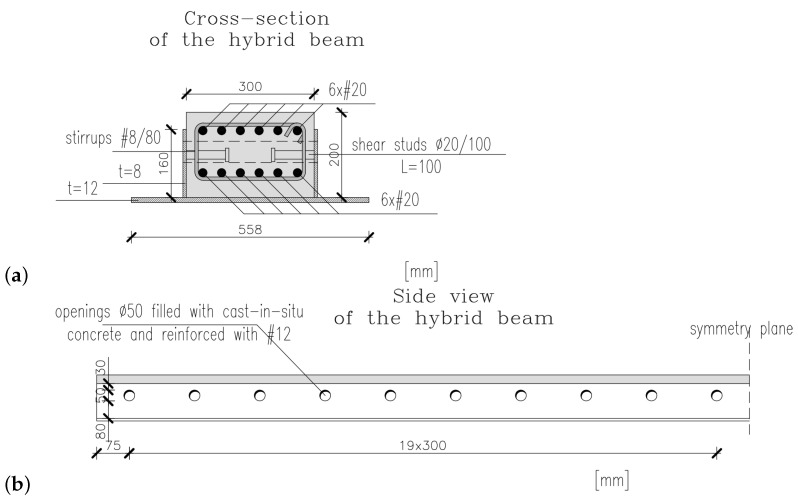
Tested hybrid beam: (**a**) cross-section, (**b**) side view.

A cross-section and a side view of the hybrid beam is shown in Figure 3. The dimensions of its RC part are 300 mm × 200 mm (width × depth) and is reinforced with 2 × 6 top and bottom rebars of 20 mm diameter. The RC core is integrated with steel profile through shear studs of diameter 20 mm spaced every 100 mm. The openings in HB are of diameter 50 mm and spaced every 300 mm. The separated beams were also tested in the ITB laboratory. The results of these tests can be found in papers [24,29]. It is worth mentioning that the longitudinal reinforcement is needed to provide the necessary fire resistance, which is consistent with the observations made by other research teams [20].

Before full-scale tests, the actual cubic compressive strength was determined for the hybrid beam (its mean value $f_{cm,cube}=85.1$ MPa) and for the infill concrete ($f_{cm,cube}=25.8$ MPa). These values basically correspond to the assumed concrete classes.

**Figure 4:**
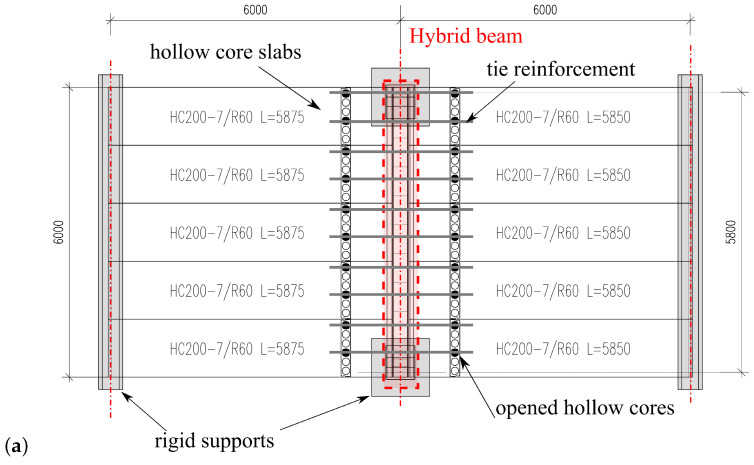

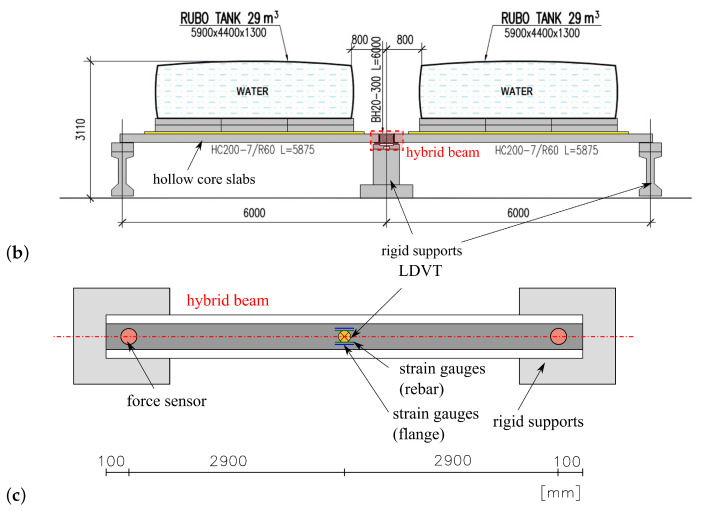
Experimental setup: (**a**) top view, (**b**) side view, (**c**) measurement devices.

**Figure 5:**
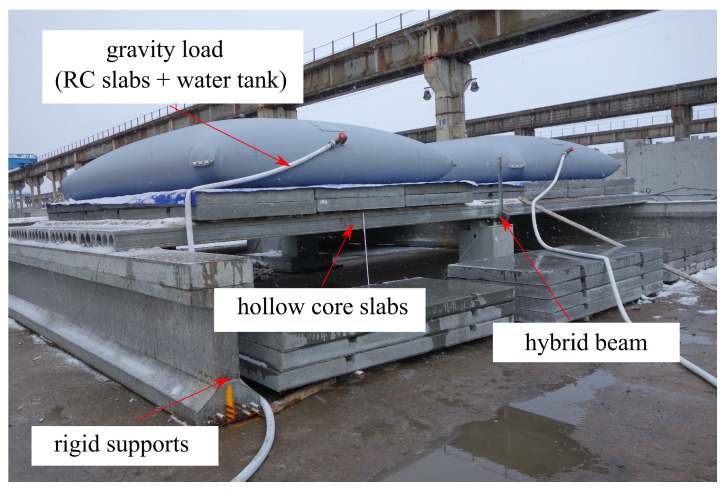
Experimental research in progress.

Top and side views of the test setup as well as measuring devices are shown in Figure 4. The hybrid beam was simply supported in two points, and the hollow core slabs were supported continuously on a central BH beam and externally on two undeformable girders. The slab system was loaded with two layers of RC plates through a layer of sand at the first stage, and later with the water poured into tanks of 29 $m^{3}$ volume. During the test, the force reactions, the midspan deflection and strains on the beam’s surfaces and the longitudinal reinforcement were measured, cf. Figure 4c. The system was not tested up to a failure, but up to the service load level. A picture of the test setup during the experimental research can be found in Figure 5.

## 3. Analytical Calculations of the Structural System Load Capacity

Checking the composite beam after casting the filler concrete requires taking into account the zones of its interaction with the floor slabs. Prefabricated pre-stressed hollow core floor slabs supported on lower flanges of the steel part of the composite BH beam with reversed TT cross-section provide a supplementary compression zone of the upper parts of an integrated floor structures.

**Figure 6:**
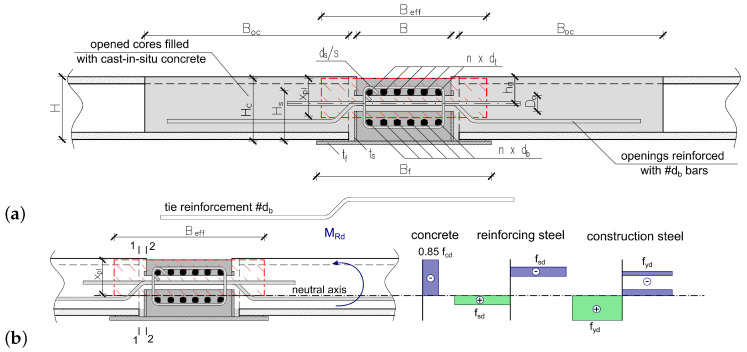
Computational models of the composite beam with interacting elements: (**a**) set of the composite floor system, (**b**) effective cross-section.

As the composite beam interacts with the adjacent reinforced concrete floor slabs, the bearing capacity of the composite beam is enhanced by expanding of the compressive zone of the concrete area (see Figure 6b). The ultimate capacity of the composite beams in the operating condition can be calculated for the slab-beam floor system due to the limit state conditions determined according to EN 1994-1-1 [30]. The selection of this approach was supported by the results of full scale tests of slab-beam floor systems.

The effective cross-section of slabs interacting with the composite beam is determined as follows. The depth of the compression zone of slab-beam floor system $x_{pl}$ (see Figure 6a) is determined according to EN 1994-1-1 [30] based on the equilibrium equation of the forces in the cross-section of the composite beam taking into account the additional forces carried out by the compression zone of the effective cross-section of the floor slabs interacting with the beam (Figure 3a). The effective flange width, $B_{eff}$ (see Figure 6), is defined by the equilibrium equation of the shearing forces resulting from longitudinal tangential stresses acting along the slab-beam joint in section 2-2 (see Figure 6b) and the compressive forces carried out by the additional compression zone of the effective cross-section of the floor slabs interacting with the composite beam, depending on the concrete compressive strength, according to the equation as follows:

(1) $$\Delta F_{d}=0.5\left(B_{eff}-B\right)\lambda \eta x_{pl}\frac{f_{ck}}{\gamma_{c}}\;\;,$$

where

$\Delta F_{d}$—the compressive forces carried out by the effective compressive zone of the effective cross-section of the floor slabs interacting with the composite beam;
*B*—the width of RC core, cf. Figure 6;
λη=0.85 (according to EN 1994-1-1 [30]);
$f_{ck}$—the characteristic compressive strength of the composite beam’s concrete;
$\gamma_{c}$—the partial safety factor for concrete. 

Therefore, the compressive forces carried out by the additional compression zone of the effective cross-section of the composite beam interacting with the floor slabs must be balanced by the tangential forces acting along the slab-beam joint, which consists of the following forces transmitted by the particular connecting elements in the considered joint (denoted as: $F_{RdiL}$, i=1,2,3,4).

- The clamping force transmitted through the indented construction joint with notches (i=1) is calculated as follows:
  (2) $$F_{Rd1L}=n_{bv}v_{Rk1}h_{bv}b_{bv}\;,$$
  where
    $v_{Rk1}=2f_{ctm}$—the longitudinal shear stress.
  $n_{bv},h_{bv},b_{bv}$—effective number, depth and width of indentations, respectively, $n_{bv}=0.5\frac{L_{1/2}}{b_{bv}}$.
    After substitution $f_{ctm}=2.2$ MPa (assuming fillet concrete C20/25), $L_{1/2}=5.80/2=2.9$ m, $h_{bv}=40$ mm, one obtains $F_{Rk1L}=255.2$ kN.
- The force transmitted by the RC dowels (i=2)
  (3) $$F_{Rd2L}=n_{d2}\;\left(A_{d}-A_{sq1}\right)\;\frac{v_{Rk1}}{\gamma_{c}}+\frac{A_{sq1}f_{sk}}{\sqrt{3}\gamma_{s}}\;,$$
  where
    $n_{d2}$—number of RC dowels per $L_{1/2}$ length;
  $v_{Rk1}=2f_{ctm}$—the longitudinal shear stress;
  $A_{d}$—the web opening area inside view (ϕ 50 mm);
  $A_{sq1}$—the reinforcement area in the opening (ϕ 12 mm);
  $f_{sk}$—the yield strength of the joint stitching reinforcement;
  $\gamma_{s}$—the partial safety factor for steel.
    After substitution $f_{ctm}=2.2$ MPa, $f_{sk}=500$ MPa, $n_{d2}=5$, $A_{d}=1963.5$ ${\mathrm{mm}}^{2}$, $A_{sq1}=113.10$ ${\mathrm{mm}}^{2}$, $\gamma_{c}=\gamma_{s}=1.0$, one obtains $F_{Rk2L}=204.0$ kN.
- The cohesive forces in the contact zone of the slab with the beam in the lower part of the beam (i=3), determined in accordance with point 6.2.5 of Eurocode 2: EN 1992-1-1 [31]. The standard coefficients *c* and μ are determined according to the requirements of EN 1992-1-1 [31], clause 6.2.5(2), allowing for the surfaces classified as very smooth, smooth, etc., as shown in the following examples:
  - Very smooth: obtained in steel, plastic or specially prepared wooden moulds; c=0.25 and μ=0.5;
  - Smooth: obtained in slip forms or as extruded or untreated surface (after vibration); c=0.35 and μ=0.6.
  However, it was found that the actual surface conditions of the steel webs in the composite beams neither correspond to the case of a smooth or very smooth surface, so it was assumed that the intermediate conditions take place, assuming the values of the coefficients: c=0.3 and μ=0.55. On this basis, the force in the contact zone between the steel web and the infill concrete was determined:
  (4) $$F_{Rd3L}=c\;\frac{f_{ctm}}{\gamma_{c}}L_{1/2}\;x$$
  where
  *x*—the depth of the compression zone of the lateral cross-section;
  $A_{sq1}=113.10$ ${\mathrm{mm}}^{2}$—the cross-sectional area of the joint stitching reinforcement spaced along the axes of the side openings in the beam;
  $d=H-h_{o}$—the effective depth, see Figure 6a.
  Other dimensions are marked in Figure 6a. On this basis, using the previously given material data, x=17.3 mm was determined. After substitution particular values into formula (4), one obtains $F_{Rk2L}=33.1$ kN.
- The friction force due to the action of compressive stresses caused by the contact force on the joint surface, equivalent to the tensile force in the joint stitching reinforcement (i=4), can be determined from the following formula:
  (5) $$F_{Rd4L}=\mu \;n_{d2}A_{sq1}\frac{f_{sk}}{\gamma_{s}}$$
  where
  $A_{sq1}$—the effective cross-sectional area of the joint stitching reinforcement;
  $n_{d2}$—number of RC dowels per $L_{1/2}$ length.
  Assuming as above μ=0.55, $n_{d2}=5$, $A_{sq1}=113.1$ ${\mathrm{mm}}^{2}$, $f_{sk}=500$ MPa, $\gamma_{s}=1.0$, one obtains $F_{Rk4L}=155.5$ kN.

Therefore, the total shear force along half of the span of the beam ($\sum_{i}F_{RdiL}$) will be equal to 647.8 kN.

The double (two-sided) shearing forces determined in this way are used in the equilibrium equation of the forces in the cross-section to determine the depth of the compression zone, and then the load-bearing capacity of the equivalent cross-section of the composite beam with the effective width of the compression zone, assuming that the strength of concrete in this zone is equal to the strength of concrete in the precast slabs. Such is the assumption of the concrete’s strength that, in the slabs’ parts interacting with the beam, the resistance of the composite cross-section is not affected, since the equilibrium equations of the section include the value of the longitudinal shearing force (647.8 kN) and not the compressive strength of the slabs’ parts. After determining the depth of the compression zone, the effective width of the parts of the plates interacting with the beam is additionally determined based on the Equation (1). On this basis, the bending load-bearing capacity of the structural floor was determined: $M_{Rd,u}=622.20$ kNm, and the total failure load of the floor: $F_{ult}=1183.74$ kN. It is worth mentioning that the presented algorithm has been calibrated using the results of various field studies, also not yet published.

## 4. Numerical Analysis

### 4.1. Description of Numerical Model

The detailed 3D FE model of the analysed system is prepared in the Abaqus environment [27]. Due to the symmetry of the hybrid beam, one-quarter of the ceiling system is modelled. The following finite elements types are used for different parts of the slim-floor system (symbols according to the programme documentation [27]):

- RC core of the hybrid beam—eight-noded continuum elements with selective integration with a default dimension of 1.5 cm—C3D8;
- Steel profile of the hybrid beam—four-noded shell elements with reduced integration with a default dimension of 2 cm—S4R;
- Shear studs—beam elements of moderate thickness and linear approximation of displacement field with a default dimension of 1.5 cm—B31;
- Rebar—two-noded truss elements with dimension adjusted to surrounding medium—T3D2,
- Hollow core slabs—six-noded wedge continuum elements with a default dimension of 3 cm—C3D6;
- Concrete filling—eight-noded continuum elements with selective integration with a default dimension of 1.5–3.0 cm—C3D8;
- Interfaces between different parts of the model—eight-noded cohesive elements—COH3D8.

The elements’ dimensions were determined through the preliminary mesh dependency studies, whose main results are shown later.

In this paper, cohesive elements were used instead of more common contact elements. They are able to correctly reproduce the concrete-to-concrete interfaces [28] as well as being much more numerically efficient due to the lack of contact pairs detecting algorithms. The Abaqus code enables users to apply viscous regularisation for cohesive elements which makes the incremental-iterative process very stable even for the range of significant delamination.

**Figure 7:**
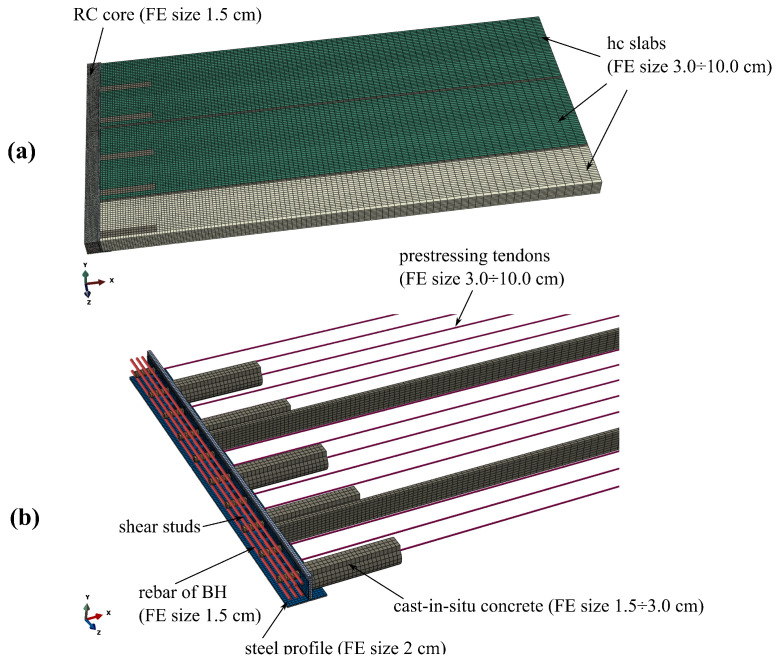
FE mesh: (**a**) the whole model, (**b**) HC slabs and RC core hidden.

**Figure 8:**
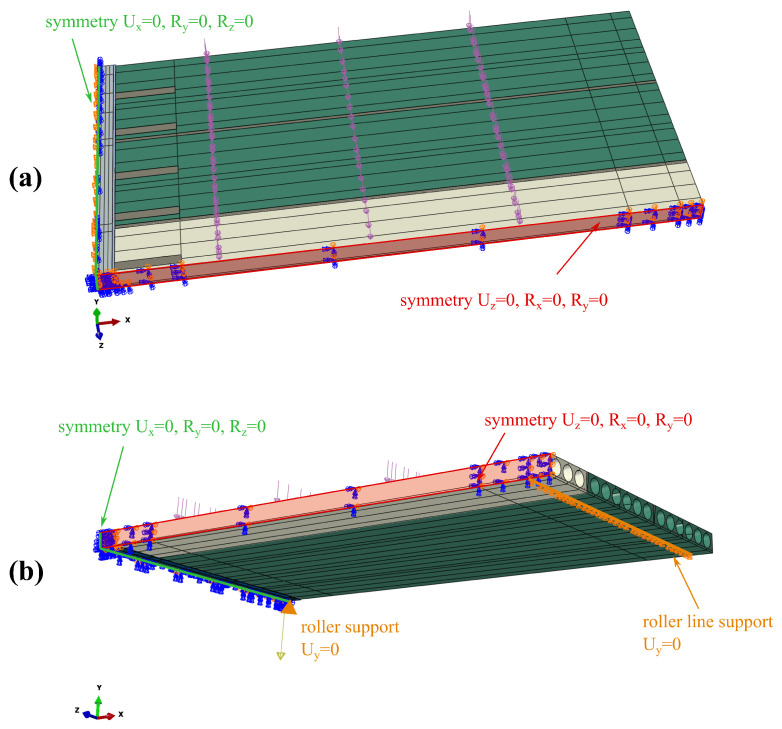
Assumed boundary conditions: (**a**) view for the top, (**b**) view from the bottom.

**Figure 9:**
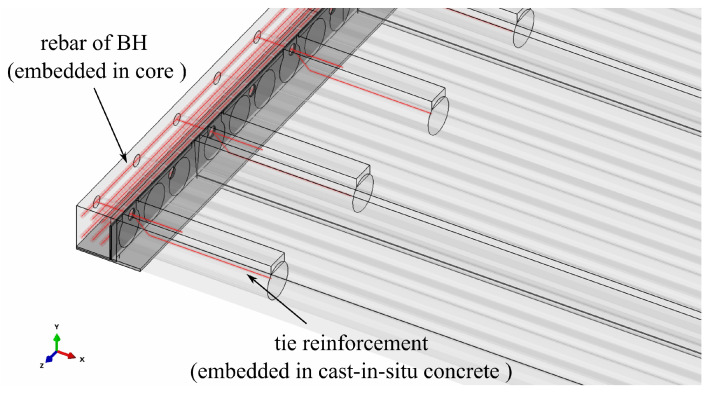
The position of rebars in the model.

**Figure 10:**
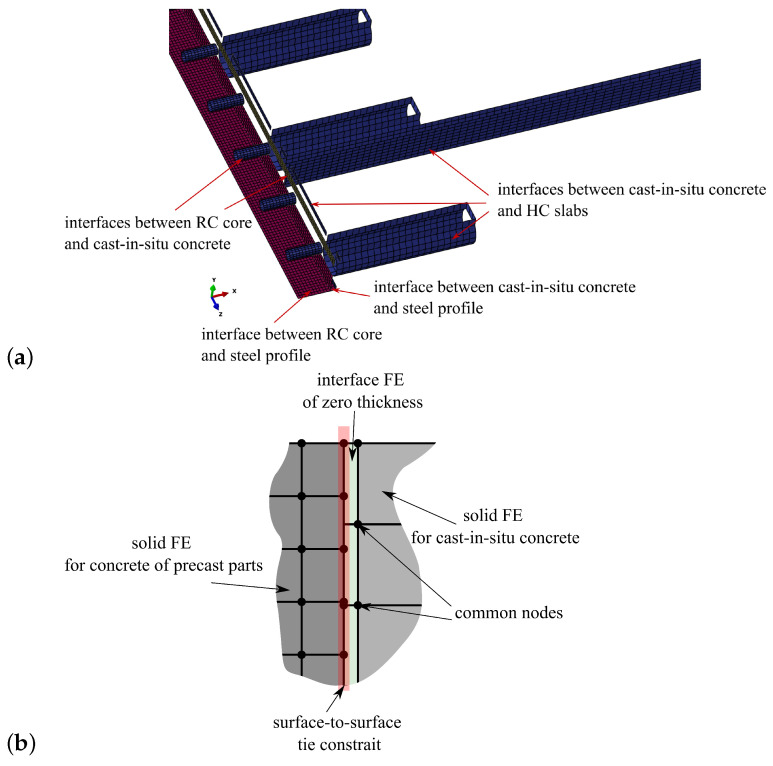
(**a**) Interfaces applied in the FE model. (**b**) Close-up of the connection between different parts.

Finite element discretisation results in more than 155 thousand elements and more than 165 thousand nodes. The FE mesh is shown in Figure 7. The boundary conditions applied in the model are as follows: for symmetry plane appropriate displacements and rotations are blocked, in the places of simple supports, vertical displacements are set to zero. The model view with the assumed boundary conditions is shown in Figure 8. The location of the rebars is depicted in Figure 9. The reinforcement was connected with the surrounding concrete part using ”Embedded” constraints, which assumes the full compatibility of the displacement field for these two parts [27,32]. The cohesive elements with zero thickness were generated on the surfaces of the parts representing cast-in situ concrete, see Figure 10a. Finite element nodes were common for these two parts. From the other side, they were connected to prefabricated parts using the ”tie surface-to-surface” option. This connection is shown schematically in Figure 10b.

The load is applied in two steps:

- The first one—a gravity load;
- The second one—a pressure to the surfaces, on which the RC plates and the water tanks were placed during the tests.

### 4.2. Constitutive Models and Their Parameters

For concrete areas, the Concrete Damaged Plasticity model (CDP) available in the Abaqus code is used in its simplified elastoplastic version (without a scalar damage parameter) since only the monotonic load path is analysed. This model has gained a lot of popularity due to its versatility and its calibration process is the subject of many scientific papers [33,34]. The paper by Lubliner et al. [35], with the modifications proposed by Lee and Fenves [36] provide the theoretical basis of this model.

The CDP model is formulated within the framework of the small-strain plasticity theory with an additive decomposition of the strain tensor ($\epsilon_{ij}$):

(6) $$\epsilon_{ij}=\epsilon_{ij}^{el}+\epsilon_{ij}^{pl}\;,$$

where $\epsilon_{ij}^{el}$—components of the elastic (reversible) strain tensor, $\epsilon_{ij}^{pl}$—components of the plastic (irreversible) strain tensor.

Thus, the stress–strain relation can be written as follows:

(7) $$\sigma_{ij}=D_{ijkl}\left(\epsilon_{kl}-\epsilon_{kl}^{pl}\right)\;,$$

where $D_{ijkl}$—components of the elasticity tensor for isotropic materials.

The material effort is measured with the Lubliner yield criterion:

(8) $$f\left(\sigma_{ij},\epsilon^{pl,c},\epsilon^{pl,t}\right)=\frac{1}{1-\alpha }\left(q-3\alpha \;p+\beta \left(\epsilon^{pl,c},\epsilon^{pl,t}\right)\langle {\hat{\sigma }}_{max}\rangle -\gamma \langle -{\hat{\sigma }}_{max}\rangle \right)-{\bar{\sigma }}^{c}\left(\epsilon^{pl,c}\right),$$

where

$\epsilon^{pl,c},\epsilon^{pl,t}$—the equivalent plastic strains in tension and compression, respectively;
α—the coefficient depending on the ratio of the initial yield strength in uni- and biaxial compressive stress states;
$p=\frac{1}{3}\sigma_{ii}$—the hydrostatic stress,
$q=\sqrt{\frac{3}{2}s_{ij}\;s_{ij}}$—the Huber–Mises equivalent stress ($s_{ij}$—components of the stress deviator);
$\beta \left(\epsilon^{c,pl},\epsilon^{t,pl}\right)=\frac{\sigma^{c}\left(\epsilon^{pl,c}\right)}{\sigma^{t}\left(\epsilon^{pl,t}\right)}\left(1-\alpha \right)-\left(1+\alpha \right)$—the function;
$\langle x\rangle =\frac{1}{2}\left(x+|x|\right)$—Macauley’s brackets, i.e., operation, which for negative variables return 0, and for positive ones, their values;
${\hat{\sigma }}_{max}$—the value of the maximal principal stress;
$\sigma^{c}\left(\epsilon^{pl,c}\right)$, $\sigma^{t}\left(\epsilon^{pl,t}\right)$—hardening/softening functions for compressive and tensile stress states, respectively;
γ—the function which controls the shape of the deviatoric section.

For yielding, the non-associated flow rule is assumed:

(9) $$d\epsilon_{ij}^{p}=d\lambda \frac{\partial G}{\partial \sigma_{ij}}.$$

The plastic potential is of smoothed Drucker–Prager type:

(10) $$G\left(\sigma_{ij}\right)=\sqrt{{\left(e\;f_{t}\;\mathrm{tg}\psi \right)}^{2}+q^{2}}-p\;\mathrm{tg}\psi ,$$

where *e*—the eccentricity parameter, which controls the smoothing rate in the vicinity of the cone apex, ψ—the dilatancy angle, $f_{t}$—the uniaxial tensile strength.

The equivalent plastic strains are calculated according to the following formulas:

(11) $$d\epsilon^{pl,t}=r\left({\hat{\sigma }}_{i}\right)\;d{\hat{\epsilon }}_{max}^{pl}$$

(12) $$d\epsilon^{pl,c}=-\left(1-r\left({\hat{\sigma }}_{i}\right)\right)d{\hat{\epsilon }}_{min}^{pl},$$

where

${\hat{\sigma }}_{i}$—values of principal stresses;
$r\left({\hat{\bar{\sigma }}}_{i}\right)=\frac{1}{2}\left(1+\frac{{\hat{\bar{\sigma }}}_{1}+{\hat{\bar{\sigma }}}_{2}+{\hat{\bar{\sigma }}}_{3}}{|{\hat{\bar{\sigma }}}_{1}\left|+\right|{\hat{\bar{\sigma }}}_{2}\left|+\right|{\hat{\bar{\sigma }}}_{3}|}\right)$—the effective stress domain function assessing “the stress state triaxiality”;
$d{\hat{\epsilon }}_{max}^{pl}$, ${\hat{\epsilon }}_{min}^{pl}$—the greatest and the smallest eigenvalues of the plastic strain rate tensor.

Due to material instability induced by the strain-softening and the non-associated flow rule, and as a consequence, the possible pathological mesh dependency of the results, the viscous Duvaut–Lions regularisation is used [33,37].

The calibration strategy has already been presented in the paper [28] and is summarised in Table 1.

**Figure 11:**
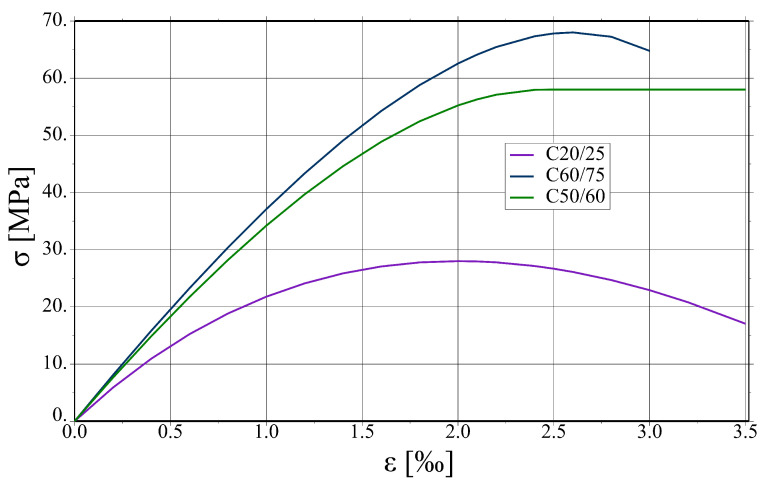
Stress (σ)–strain (ϵ) curves for concretes of different classes in uniaxial compression.

**Figure 12:**
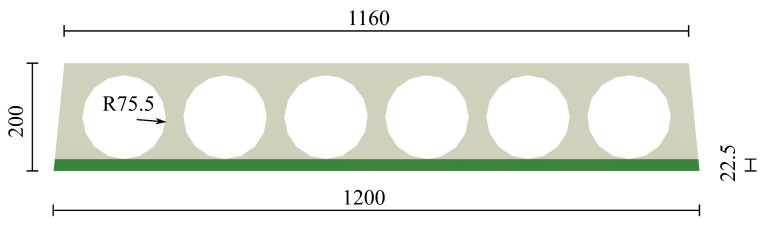
Cross section of a hollow core slab. The zone with the increased tensile strength is marked in green.

The parameters assumed for concrete of different classes are summarised in Table 2. In the compression regime, the function from EC2 [31], sometimes called ”Madrid parabola”, is assumed, see Figure 11. The method of modelling prestressed plates requires some comment. In the analysed slab system, prestressed HCS are connected with hybrid beams in an almost rigid way. Consequently, the static scheme of the whole system is not statically determined, so introducing prestressing forces in tendons would cause additional stresses in parts other than HCS. In the real world, HCS are prestressed in prefabrication plants, then placed in a slab system, and finally, the connection is made after pouring the filler concrete. Taking into account these stages in fully physically justified manner is a very complex task. Moreover, the hybrid beams’ vicinity is the main area of interest in the present studies. Therefore, some simplification concerning HCS is made. It is assumed that near tendons, cf. green area in Figure 12, the tensile strength is increased by the value of prestressing stress and that the behaviour of HCS concrete is more ductile than the other parts (no descending branches in tension and compression).

**Figure 13:**
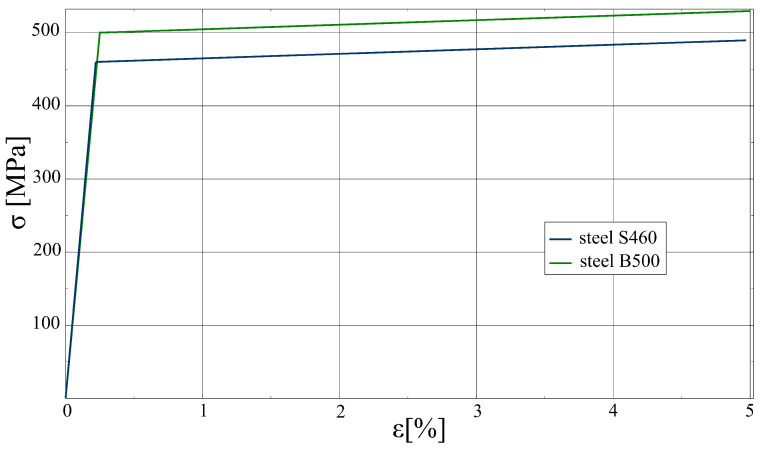
Stress (σ)–strain (ϵ) curves for steel of different grades in uniaxial stress state.

For steel regions, an elastoplastic constitutive relation with the Huber–von Mises yield surface, associated flow rule, and kinematic, linear hardening is assumed ($f_{y}$—the yield strength; $E_{T}$—the hardening modulus). The parameters for different steel grades were assumed on the basis of Eurocode 2 [31] and Eurocode 3 [40] and are summarised in Table 3. The stress–strain curves for steels in the uniaxial stress state are shown in Figure 13. For prestressing tendons, a linear elastic model is adopted.

In the case of interface elements, the traction–separation law available by default in the Abaqus code is used with modifications proposed in paper [28]. It is formulated in the framework of damaged elasticity. In the elastic regime, the traction–separation law has the following form:

(13) $$t=\left[\begin{array}{c} t_{n}\\ t_{s}\\ t_{t} \end{array}\right]=\left[\begin{array}{ccc} K_{n} & 0 & 0\\ 0 & K_{s} & 0\\ 0 & 0 & K_{t} \end{array}\right]\;\left[\begin{array}{c} \epsilon_{n}\\ \epsilon_{s}\\ \epsilon_{t} \end{array}\right]=K\;\epsilon ,$$

where

t—the tractions vector; indices *n*, *s*, and *t* refer to the normal, first, and second tangential direction, respectively;
$K_{n}$, $K_{s}$, $K_{t}$—the stiffness of interface material in three directions;
$\epsilon_{n}=\frac{\delta_{n}}{T_{0}}$, $\epsilon_{s}=\frac{\delta_{s}}{T_{0}}$, $\epsilon_{t}=\frac{\delta_{t}}{T_{0}}$—interface nominal strains;
$T_{0}$—the initial thickness of the interface;
$\delta_{n}$, $\delta_{s}$, $\delta_{t}$—separations (displacement jumps at an interface) in three directions.

The elastic stiffness for concrete–concrete interfaces has no clear physical meaning, since this kind of interface can be classified as rigid before the damage initiation. Nonetheless, the assumption of a very small interface thickness, resulting in large values of its stiffness, can lead to convergence problems due to the large values of unbalanced forces. On the other hand, introducing too small values can reduce stress concentration effects, which can occur in the vicinity of the interface boundaries. Consequently, the values deduced from the ATENA manual appear to be a reasonable choice [41]:

(14) $$T_{0}\approx 0.005\;a,$$

where *a*—the biggest dimension of the connected parts.

The damage initiation criterion controls the onset of the damage state [42]. The quadratic nominal stress criterion with the modification described in-detail in [28] is selected in this study. It can be expressed as follows:

(15) $${\left(\frac{\langle t_{n}\rangle }{f_{ctm,int}}\right)}^{2}+{\left(\frac{t_{s}}{\tau^{\prime ult}\left(t_{n}\right)}\right)}^{2}+{\left(\frac{t_{t}}{\tau^{\prime ult}\left(t_{n}\right)}\right)}^{2}=1,$$

where

$f_{ctm,int}$—the tensile strength of the interface;
$\tau^{\prime ult}\left(t_{n}\right)=\left\{\begin{array}{cc} f_{sh} & \mathrm{if}\;t_{n}\geq \;0\\ \tau^{ult}\left(t_{n}\right) & \mathrm{if}\;t_{n}<0 \end{array}\right.$
$\tau^{ult}\left(t_{n}\right)=\sqrt{{\left(c-t_{n}\tan\left(\phi \right)\right)}^{2}-{\left(c-f_{ctm,int}\tan\left(\phi \right)\right)}^{2}}$—Carol’s formula [43,44];
*c*, ϕ—the cohesion and the friction angel, respectively;
$f_{sh}$—the shear strength.

**Figure 14:**
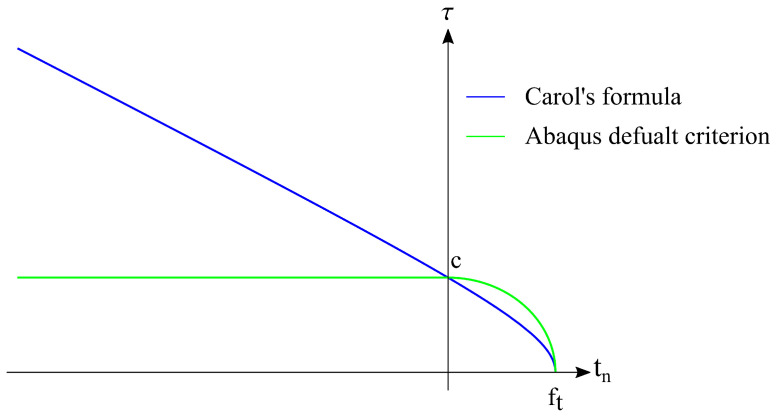
The modified damage initiation criterion.

It should be stated that the modification of the default Abaqus criterion concerns taking into account the increase in shear strength due to compressive normal traction. The USDFLD user’s subroutine is used to modify this feature of the interface model. The standard and modified criterion is shown in Figure 14. Normal traction to the interface ($t_{n}$) is marked on the horizontal axis, and the resultant shear traction (τ)—on the vertical axis. No-damage traction states are located inside the envelope.

After the tractions exceed the damage-initiation criterion, they are calculated taking into account the damage factor *D*:

(16) $$\begin{array}{c} t_{n}=\left\{\begin{array}{ccc} \left(1-D\right)K_{nn}\;\epsilon_{n} & \mathrm{if} & \epsilon_{n}\geq 0\\ K_{nn}\;\epsilon_{n} & \mathrm{if} & \epsilon_{n}<0 \end{array}\right.\\ t_{s}=\left(1-D\right)K_{ss}\;\epsilon_{s}\\ t_{t}=\left(1-D\right)K_{ss}\;\epsilon_{t} \end{array}$$

As one can notice, the damage factor is taken into account in the case of shear and normal tensile tractions and is neglected for normal compressive traction. The evolution of the damage parameter under a combination of normal and shear deformation is ruled by the effective separation as follows:

(17) $$\delta_{eqv}=\sqrt{{\langle \delta_{n}\rangle }^{2}+\delta_{s}^{2}+\delta_{t}^{2}}\;.$$

In the present study, the exponential damage evolution law is used:

(18) $$D\left(\delta_{eqv}^{max}\right)=1-{\left(\frac{\delta_{eqv}^{init}}{\delta_{eqv}^{max}}\right)}^{2}\left(1-\frac{1-\exp\left(-\alpha \frac{\delta_{eqv}^{max}-\delta_{eqv}^{init}}{\delta_{eqv}^{fail}-\delta_{eqv}^{init}}\right)}{1-\exp(-\alpha )}\right),$$

where

**Figure 15:**
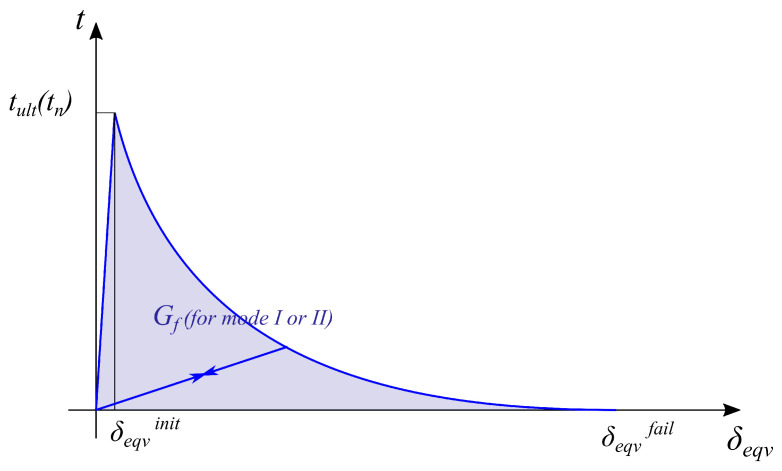
Plot of the traction–separation law with basic parameters marked.

$\delta_{eqv}^{max}$—the maximum effective separation during the loading history;
$\delta_{eqv}^{init}$—the effective separation at the damage initiation;
$\delta_{eqv}^{fail}$—the effective separation at complete failure;
α—the parameter which controls the rate of softening (typical value is around 7 cf. [28]). 
The behaviour of the model, i.e., tractions—equivalent separations, are presented in Figure 15.

The failure separation is calculated on the basis of fracture energy $G_{f}$ [28]. The value of energy dissipated during the separation of the concrete-to-concrete interface differs significantly for tension and shear states. In the present study, the effective displacement at failure was related to the fracture energy for the mode I type of fracture ($G_{f,I}$) or mode II ($G_{f,II}$). The fracture mode is distinguished from the dominant mode share in the total strain energy. The approach to calculating the fracture energy value is described in the paper [28] together with its justification. The value of interface fracture energy is related to the bond efficiency coefficient and the fracture energy of weaker concrete. The bond efficiency coefficient is defined in the following manner [45]:

(19) $$\alpha_{int}=\frac{f_{ctm,int}}{f_{ctm,weak}},$$

where $f_{ctm,int}$ and $f_{ctm,weak}$ are the tensile strength of the interface and the weaker concrete layer, respectively. It is worth mentioning that the bond efficiency coefficient in the case of large-scale structures like composite slabs can be significantly reduced due to shrinkage strains.

The calibration strategy is summarised in Table 4, whereas parameters correspond to different types of interfaces in Table 5. For the interface between the RC part of HB and infill concrete, the shear strength and the fracture energy in mode II are increased in order to take into account impact of indentations.

### 4.3. Results of the Analysis

Before main calculations, mesh dependency studies and a parametric analysis concerning viscous regularisation were performed to verify the correctness of the results. Firstly, three meshes were analysed:

- Coarse: 42 k finite elements (FE) and 139 k degrees of freedom (D.O.F), representative FE dimension for the RC core—2.2 cm;
- Default: 158 k FE and 472 k D.O.F.; representative FE dimension for the RC core—1.5 cm,
- Fine: 233 k FE and 656 k D.O.F., representative FE dimension for the RC core—0.8 cm.

**Figure 16:**
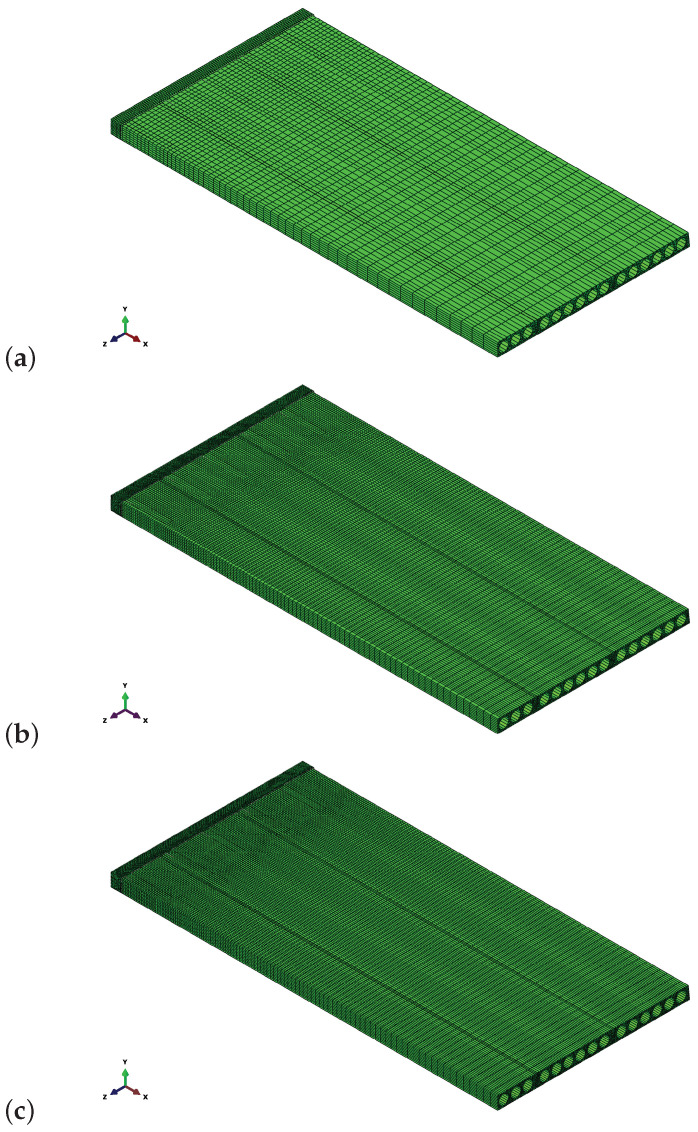
Meshes adopted in the mesh dependency studies: (**a**) coarse—139 k D.O.F, (**b**) default—472 k D.O.F, (**c**) fine—656 k D.O.F.

**Figure 17:**
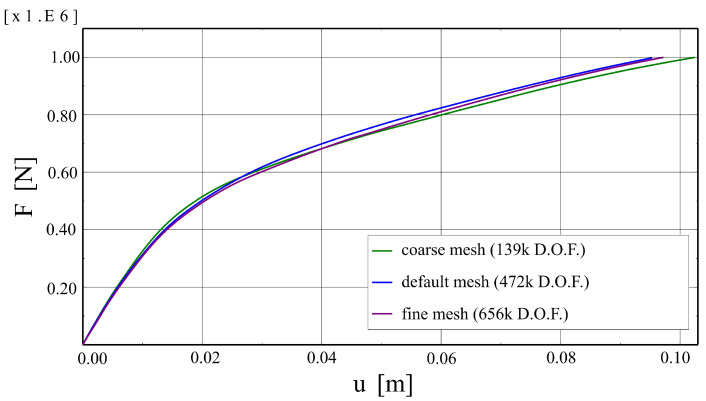
Comparison of force–deflection curves obtained with various meshes.

The analysed meshes are shown in Figure 16. Results obtained for them are summarised in Figure 17 as force–deflection curves. The plots are almost the same, so the default mesh density can be considered sufficient.

**Figure 18:**
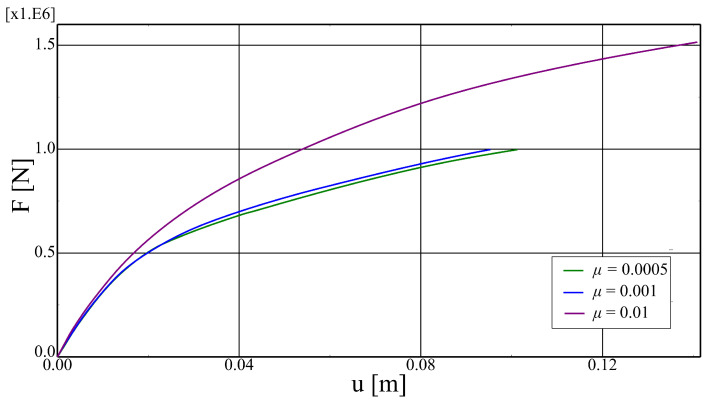
Comparison of force–deflection curves obtained for different values of viscous parameter μ.

Some parameters of the applied constitutive models have no clear physical meaning, especially the viscous parameter μ, which is used to obtain a stable incremental-interactive process. Therefore, the parametric analysis concerning μ was performed. Three values of this parameter were analysed, namely: 0.0005, 0.001, 0.01. The results are shown in Figure 18 in the form of force–deflection plots. The higher the value of the viscous parameter, the higher the stiffness of the system is predicted by the model. However, the results obtained for the two smaller values are very similar. Consequently, the model can be considered not burdened with significant numerical errors.

**Figure 19:**
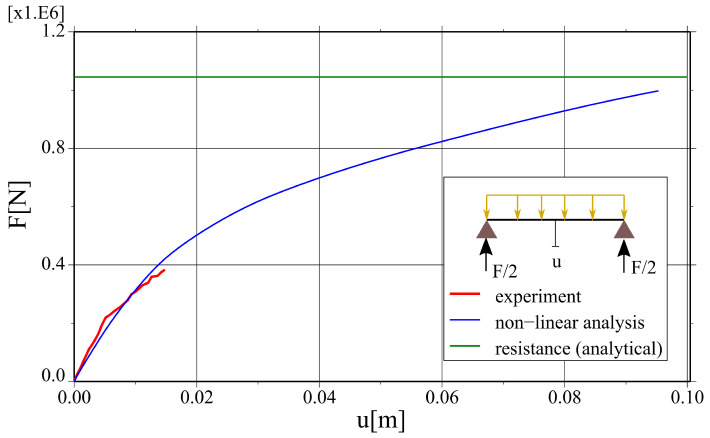
Comparison of force–deflection curves obtained from experimental tests and non-linear FE analysis.

**Figure 20:**
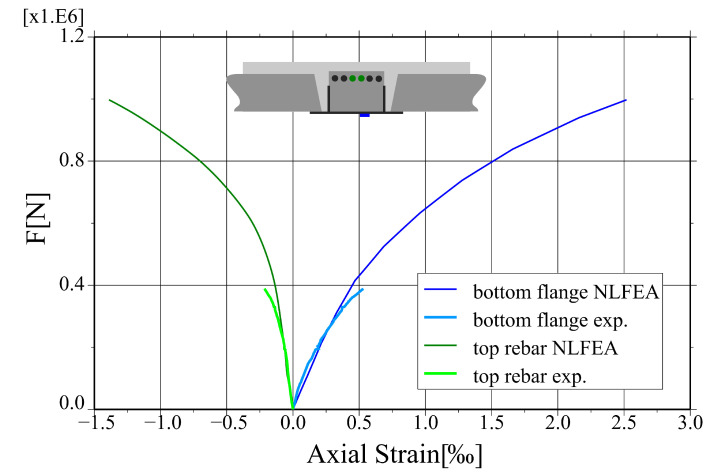
Comparison of strains plot obtained from the non-linear analysis and for the experimental tests.

The results of the non-linear finite element analysis are confronted to the results of experimental tests and analytical calculations in Figure 19 as a total force—mid-deflection plot. The numerical values and their relative differences are summarised in Table 6. The relative error of reaction caused by self-weight is 11.3%, which can be caused, e.g., by an uneven distribution of reactions in the whole structural system. The relative difference in the load capacity estimation between non-linear analysis and analytical calculations is 3.9%. The measured and calculated axial strains for the bottom flange and the top rebar in the middle of the span are compared in Figure 20. The initial stiffness of the system is correctly captured by the numerical model, and some discrepancies start to occur for the higher load values—the relative difference between results of the FE analysis and the field tests is 12.8% for the service load level. All in all, keeping in mind the complexity of the analysed structural system, the agreement between the results of different analyses is fairly satisfactory.

**Figure 21:**
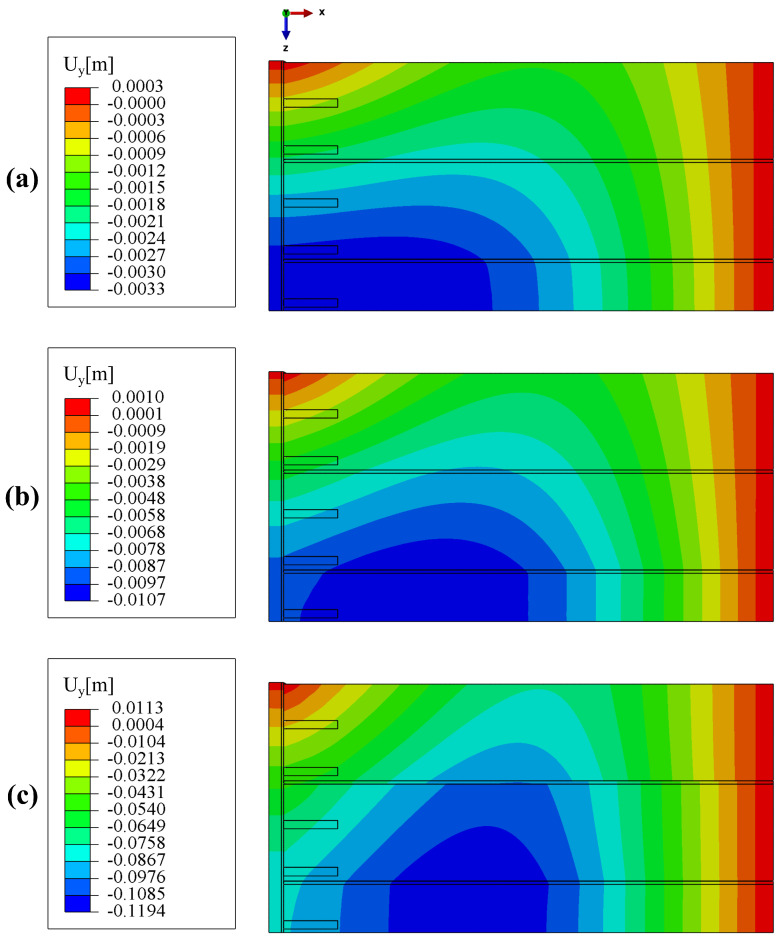
Maps of the slab deflection for values of total force: (**a**) 138 kN (dead weight), (**b**) 349 kN, (**c**) 978 kN.

Maps of the slab deflection, i.e., vertical displacement, for different load levels are shown in Figure 21. One can notice that the model is able to capture the “curling effect”, i.e., for low load levels hollow core slabs deform together, whereas for higher ones discontinuities in the displacement field are clearly visible.

**Figure 22:**
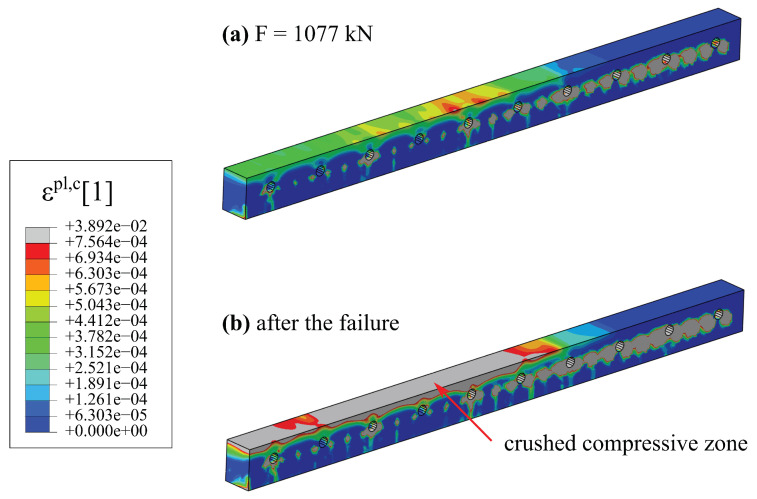
Maps of equivalent plastic strain in compression for concrete of the hybrid beam: (**a**) just before the failure F=1077 kN, (**b**) after the failure.

**Figure 23:**
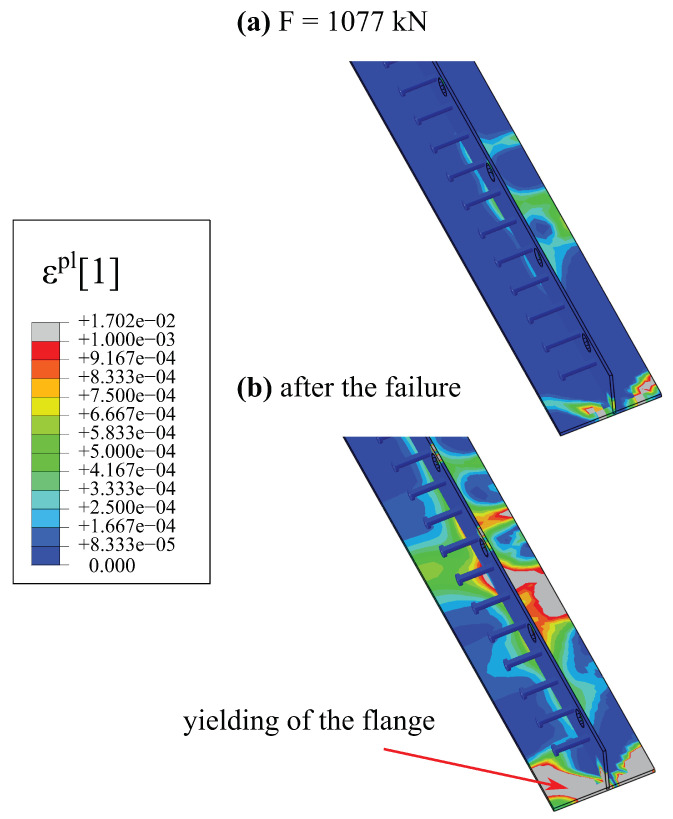
Maps of equivalent plastic strain for steel of the hybrid beam: (**a**) just before failure F=1077 kN, (**b**) after the failure.

The system is considered damaged if the compressive zone of the main load-carrying element, i.e., the hybrid beam, is crushed [24]. Figure 22 shows maps of the equivalent plastic strain in compression ($\epsilon^{pl,c}$) for two loads level: just before and just after the failure. Regions in which concrete is in softening regime are marked with grey colour. When the concrete is in softening phase, due to force plastic redistribution, the steel of the bottom flange starts to yield, which is depicted in Figure 23. The main plastic zones are also marked in grey. To sum up, the failure mode of the hybrid beam is correctly captured.

**Figure 24:**
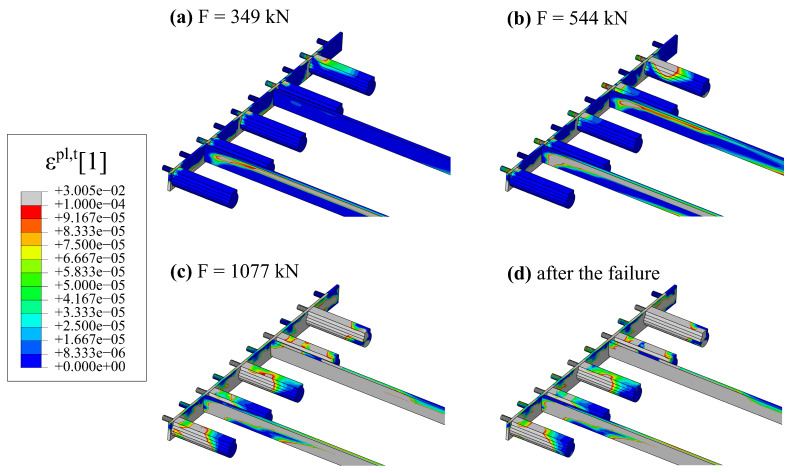
Maps of equivalent plastic strain in tension for the concrete infill: (**a**) for F=349 kN, (**b**) for F=544 kN, (**c**) just before failure F=1077 kN, (**d**) after the failure.

Longitudinal shear between the hybrid beam and the hollow core slabs is carried, among others, by RC dowels from concrete in hollow cores cast in situ. Figure 24 shows a progressive failure of this part of the structural system. One can see maps of equivalent plastic strain in tension ($\epsilon^{pl,t}$). Areas in grey colour can be considered cracked (concrete in softening phase in tension).

**Figure 25:**
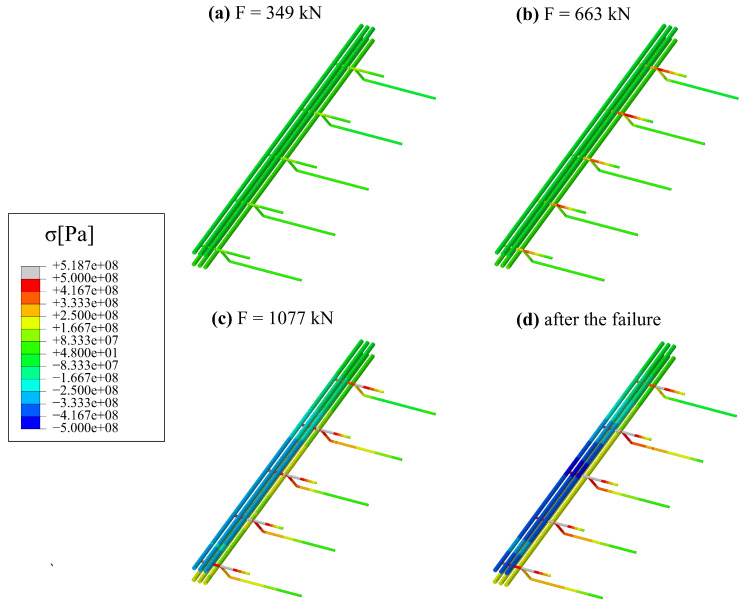
Maps of stresses in rebar: (**a**) for F=349 kN, (**b**) for F=633 kN, (**c**) just before failure F=1077 kN, (**d**) after the failure.

The stresses in the rebars for different load levels are shown in Figure 25. Up to the failure, the stresses in the longitudinal reinforcement of the beam are rather low since it is mainly needed for fire conditions. At the failure, the stresses in the top bars increased significantly due to the stress redistribution after crushing the compressive zone. The higher stress levels can be observed for the tie reinforcement of the concrete dowels.

## 5. Discussion

The process of the creation and validation of the numerical model for a new slim-floor system with composite steel–concrete hybrid beams is presented in the previous paragraphs. The numerical model was validated using experimental research results (the stiffness) and analytical calculations (the load capacity). It is crucial for the correct prediction of these characteristics of the structural system is the proper model for concrete–concrete interfaces based on cohesive elements, which was proposed in the paper [28]. The total area of the interfaces is very significant, so more sophisticated models for them are also needed.

The initial stiffness is perfectly captured by the model. Some discrepancies occur for the higher load levels. In general, the model predicts slightly too stiff response for them, which can be caused, e.g., by loading conditions. The load was applied extremely slowly due to the need to lay the loading RC slabs, and then to fill the tanks with water. This can result in the occurrence of delayed elastic response not covered by standard constitutive models. The other possible explanation is related to the asymmetry of the hollow-core slab and loading slabs arrangement. However, the assumption of symmetry planes is relevant for the reasonable computing time.

The model covers the phenomena of the “curling effect” typical for structures made of hollow core slabs and the progressive damage of connections between different parts of the system. Moreover, it is able to reproduce the failure mode of a hybrid beam—simultaneous crushing of the compressive zone and yielding of the lower flange. Additionally, the failure mode proves the economical design of the hybrid beams cross-section.

The mesh-dependency of the analysed model is rather insignificant, which is caused by the fact that the stiffness of the system is ruled by the cohesive elements. The constitutive models for the interfaces are formulated as a tractions–displacement relation, so they do not need any internal length scale parameters to obtain mesh-independent results. On the other hand, the outcomes of numerical simulations are strongly dependent on the value of the viscous parameter μ, which is needed to overcome the convergence problems typical for strain-softening models. The user can significantly reduce the computation time by using a higher value of this parameter, but as a result the behaviour of the system may turn out to be too rigid; see Figure 18.

The result of numerical studies is used during the planning of further field tests. For instance, they showed that the slim-floor ceilings should be subjected to cylindrical bending rather than basin form of deformation in order to obtain a pure bending resistance. Consequently, the external edges of the hollow-core slabs should be flexibly supported on hybrid beams. In the present static scheme, some hollow core slabs are torqued, which, in turn result in an uneven transfer of loads to the beams. In Figure 22, one can notice that the highest values of plastic strains before the failure are not located in the middle of the span, but in the vicinity of RC dowels in the openings.

## 6. Conclusions

A detailed 3D non-linear model of the new slim-floor system on hybrid beams is presented in this paper. It was validated using the results of experimental research (stiffness) and the analytical calculations (the load capacity). The model is being used during the planning of further field tests, to understand in-depth the outcomes of experimental research or to verify design formulas. Such an approach is increasingly common during the development of innovative construction products and solutions. Based on the analyses carried out, the following main conclusions can be drawn:

- In the described experimental field studies, it was proved that the new slim-floor system has the proper level of reliability. The registered deflections and strains for the service load level were not of great values. The system was almost at the elastic stage.
- The fairly good agreement between the different methods of assessments is obtained when the system and its execution complexity are taken into account. The relative difference for the ultimate force estimation is 3.9% (FE analysis vs. analytical calculations). The initial stiffness of the slim-floor system is perfectly captured by the model, whereas deflection for higher load levels is predicted with satisfactory accuracy—the relative difference for deflection under service load is 12.8% (FE analysis vs. experimenta research).
- The stiffness of slim-floor systems is ruled by the progressive loss of connection between the system parts. Therefore, a proper model of interfaces between different materials (or concretes cast at different times) is an essential issue for correct deflection predictions as well as non-linear material models for infill concrete. The approach described in [28] based on the adaptation of a standard Abaqus constitutive model for interfaces, using cohesive elements and enhanced with the user’s USDFLD subroutine strength envelope allowed us to obtain reliable results and is numerically efficient.
- Since the failure mode of the hybrid beam is not very typical for composite steel–concrete structures and consists in crushing the concrete compressive zone, a reliable model for concrete cores of hybrid beams is also of great importance. The widespread Concrete Damaged Plasticity model [27] proved its versatility and enabled the correct prediction of the failure mode and the value of the failure load for the analysed system. The calibration of its parameters is mostly based on the provisions of Eurocode 2 [31].
- Mesh dependency studies have revealed that the elements and their dimensions proposed in Section 4.1 are appropriate for such analyses. The system deformability is dependent mostly on the delamination of the interface zones. Consequently, the numerical model is not prone to mesh-dependency effects typical for strain-softening models since the constitutive models for interfaces are formulated as the traction–separation relations. On the other hand, users should be cautious in using viscous regularisation because it can cause a strong stiffening of the response of the model.

Summarising, the detailed instructions for the preparation of non-linear numerical models for the whole slim-floor structural systems were formulated within this paper. It is advised to use cohesive elements instead of contact ones due to their numerical efficiency. Rules of calibration material models for such interface elements were reported. Moreover, design formulas for the load capacity estimation of structural systems consisting of precast elements and cast-in situ concrete are explained in detail.

Further research on the topic of modern slim-floor systems is planned. Primarily, a new experimental campaign is currently being conducted, in which the entire floor systems are tested up to the point of failure. These results will be published together with the corresponding numerical models. Other strategies for numerical modelling of composite floors include, for example, the use of fibre beam elements [46,47]. Moreover, the considered slim-floor ceiling system is constantly being developed. For instance, new types of floor systems and cross-sections of hybrid beams will be analysed. These improvements will probably make the described innovative product even more sustainable.

## Figures and Tables

**Table 1:** The calibration of the CDP model—main assumptions. (*)—uniaxial strength expressed in MPa and dimensions in mm.

Symbol	Parameter	Assumed Value/Formula	Reference
	Elasticity		
*E*	Young’s Modulus	$E_{cm}=22,000{\left(f_{cm}\right)}^{0.3}$ (*)	EN 1992-1-1 [31]
ν	Poisson’s ratio	0.2	EN 1992-1-1 [31]
	Ultimate surface		
$f_{cm}$	Uniaxial compressive strength	$f_{cm}=f_{ck}+8$ (*)	EN 1992-1-1 [31]
$f_{ctm}$	Uniaxial tensile strength	$f_{ctm}=0.3f_{ck}^{2/3}$, $f_{ck}\leq 50$ MPa $f_{ctm}=2.12\;\ln\left(1+0.1f_{cm}\right)$, $f_{ck}>50$ MPa (*)	EN 1992-1-1 [31]
$\frac{f_{b0}}{f_{c0}}$	Ratio of biaxial to uniaxial compressive strength	1.16	[27]
$K_{c}$	Parameter controlling the shape of deviatoric section	0.667	[27]
	Hardening/Softening Rule		
$\sigma^{c}\left(\epsilon^{pl,c}\right)$	Hardening rule in compression	Madrid parabola	EN 1992-1-1 [31]
$\sigma^{t}\left(\epsilon^{pl,t}\right)$	Linear softening in tension ruled by fracture energy	$G_{f}=10{\left(d_{max}\right)}^{0.33}{\left(f_{cm}\right)}^{0.33}$ (*)	JSCE [38]
	Plastic Potential and Viscoplastic Regularisation		
ψ	Dilatancy angle	$30^{\circ }$	[39]
$\epsilon_{0}$	Eccentricity	0.1	[27]
μ	Viscosity parameter	0.0001	[33]

**Table 2:** Parameters assumed for concrete.

Feature	C20/25	C50/60	C50/60 Prestressed Area	C60/75
*E* [GPa]	30	37	37	39
ν [1]	0.2	0.2	0.2	0.2
$f_{cm}$ [MPa]	28	58	58	68
$f_{ctm}$ [MPa]	2.2	4.1	11.1	4.4
behaviour in tension	linear softening $G_{f}=75$ N/m	linear softening $G_{f}=95$ N/m	no softening	linear softening $G_{f}=100$ N/m

**Table 3:** Parameters assumed for steel parts.

Feature	Profile—S460	Rebar—B500	Tendons
*E* [GPa]	210	200	200
ν [1]	0.3	-	-
$f_{y}$ [MPa]	460	500	-
$E_{t}$ [GPa]	1	1	-

**Table 4:** The calibration of a traction–separation model for a concrete-to-concrete interface—main assumptions. $E_{weak}$—the Young’s modulus of weaker concrete, $G_{weak}$—the Kirchhoff’s modulus of weaker concrete.

Symbol	Parameter	Assumed Value/Formula	Reference
	Elasticity		
$K_{nn}$	Stiffness in normal direction	$E_{weak}$	[41]
$K_{ss}$, $K_{tt}$	Stiffness in tangential directions	$K_{ss}=K_{tt}=G_{weak}$	[41]
*t*	Interface thickness	t=0.005a	[41]
	Damage Initiation Criterion		
$f_{ctm}$	Tensile strength (mode I)	$f_{ctm,int}=\alpha_{int}\;f_{ctm,weak}$	[28,45]
$f_{sh}$	Shear strength (mode II)	$f_{sh}\left(t_{n}\right)$ acc. to Carol’s Formula, USDFLD	[43]
	Damage Evolution Rule and Viscous Regularisation		
	Softening type	Exponential shape	
α	Parameter of exponential function	≈7	[28]
$\delta_{m}^{fail,I}$	Failure separation in mode I	Based on the fracture energy	[28]
$\delta_{m}^{fail,II}$	Failure separation in mode II	Based on the fracture energy	[28]
μ	Viscosity parameter	0.001	

**Table 5:** Parameters assumed for interfaces.

Feature	HB Concrete— Infill	HB Steel— Infill	HCS— Infill
$\alpha_{int}$ [1]	0.5	0.25	0.32
$K_{nn}$ [GPa]	30	30	30
$K_{tt}$ or $K_{ss}$ [GPa]	12.5	12.5	12.5
$T_{0}$ [m]	0.01	0.01	0.01
$f_{ctm}$ [MPa]	1.2	0.55	0.7
*c* [MPa]	3.1	1.1	1.4
ϕ [deg]	40	40	40
$G_{f,I}$ [N/m]	20	10	13
$\delta_{eqv}^{fail,I}$ [m]	0.00012	0.00012	0.00013
$G_{f,II}$ [N/m]	1200	200	250
$\delta_{eqv}^{fail,II}$ [m]	0.0037	0.0015	0.0016

**Table 6:** Comparison of the selected results for different performance assessments methods.

	Experiment	Analytical Calculations	NLFEA	Difference [%]
self-weight	F=124 kN	n/a	F=138 kN	11.3%
load capacity	n/a	F=1183 kN	F=1137 kN	3.9%
deflection for service load	u=14.8 mm	n/a	u=12.9 mm	12.8%
(F=389 kN)				

## Data Availability

Data are contained within the article.
